# Serum Calprotectin as a Novel Biomarker of Disease Severity and Activity in Systemic Sclerosis Patients

**DOI:** 10.3390/ijms26094290

**Published:** 2025-05-01

**Authors:** Chiara Pellicano, Annalisa Villa, Valeria Carnazzo, Giancarlo D’Ippolito, Ilaria Vinante, Federica Laterza, Umberto Basile, Edoardo Rosato, Antonietta Gigante

**Affiliations:** 1 Department of Translational and Precision Medicine, Sapienza University of Rome, 00185 Rome, Italy; chiara.pellicano@uniroma1.it (C.P.); annalisa.villa@uniroma1.it (A.V.); giancarlo.dippolito@uniroma1.it (G.D.); edoardo.rosato@uniroma1.it (E.R.); 2 Dipartimento di Patologia Clinica, Ospedale Santa Maria Goretti, A.U.S.L. Latina, 04100 Latina, Italy; v.carnazzo@ausl.latina.it (V.C.); i.vinante@ausl.latina.it (I.V.); f.laterza@ausl.latina.it (F.L.); u.basile@ausl.latina.it (U.B.)

**Keywords:** systemic sclerosis, calprotectin, fibrosis, interstitial lung disease, digital ulcers, biomarker

## Abstract

A monocentric cross-sectional study was performed to investigate the role of serum calprotectin as a biomarker for disease severity and activity in systemic sclerosis (SSc). Serum calprotectin was measured in 74 consecutive SSc patients admitted to a tertiary hospital in Rome, and in 50 healthy controls (HCs) who were healthcare workers, using Aptiva’s particle-based multianalyte technology. In SSc patients, a statistically significant correlation was found between calprotectin and the modified Rodnan skin score (mRSS) (r = 0.402, *p* < 0.001), disease activity index (DAI) (r = 0.420, *p* < 0.001), disease severity scale (DSS) (r = 0.365, *p* < 0.01), forced vital capacity (FVC) (r = −0.459, *p* < 0.001), and diffusion lung capacity for carbon monoxide (DLco) (r = −0.445, *p* < 0.001). Calprotectin was higher in SSc patients with digital ulcers (DUs) than in SSc patients without DUs [2.98 mcg/mL (IQR 2.07;4.29) vs. 2.08 mcg/mL (IQR 1.71;2.45), *p* < 0.01] and in SSc patients with interstitial lung disease (ILD) compared to SSc patients without ILD [2.56 mcg/mL (IQR 1.94;3.03) vs. 1.96 mcg/mL (IQR 1.7;2.35), *p* < 0.01]. The multivariable stepwise logistic regression analysis showed calprotectin to be independently associated with DUs [OR 2.531 (CI 95%: 1.074;5.961), *p* < 0.05] and ILD [OR 3.687 (CI 95%: 1.336;10.170), *p* < 0.05] in SSc patients. Serum calprotectin is associated with DUs and ILD in SSc patients.

## 1. Introduction

Systemic sclerosis (SSc) is a complex systemic autoimmune disease characterized by pronounced vasculopathy, immune system dysregulation, and progressive fibrosis of the skin and internal organs [1]. Despite the marked pleomorphism of this disease, common clinical manifestations include skin fibrosis of varying extent, interstitial lung disease (ILD), and vascular complications, including digital ulcers (DUs) [1].

Microvascular damage, which is the hallmark of SSc, is characterized by a progressive decrease in capillary density and structural alterations detectable through the nailfold video capillaroscopy (NVC) [2].

The most common microvascular complication in SSc patients is the development of DUs, affecting about 30–40% of patients [3]. DUs, defined as any loss of epithelialization and tissues localized on the fingertip, are closely associated with reduced quality of life and may be an indicator of advanced vascular and inflammatory involvement [4]. Several distinct risk factors for the development of DUs have been identified, including a high modified Rodnan skin score (mRSS), a diffuse cutaneous (dc)SSc subset, the presence of Scl70 antibodies, or a late capillaroscopic pattern [3].

ILD is the leading cause of morbidity and mortality in SSc patients, and 25–30% of them will ultimately progress to respiratory failure or death [5]. Most patients exhibit a stable or slowly progressive disease course, characterized by a slow decline in lung function or a minimal increase in the extent of pulmonary fibrosis, as observed by high-resolution computed tomography (HRCT). SSc patients with ILD experience a progressive reduction in lung capacity, as measured by parameters such as forced vital capacity (FVC) and diffusion lung capacity for carbon monoxide (DLco). In the earlier stages of ILD, there is a reduction in DLco with preserved FVC, and HRCT shows a ground-glass pattern with typical subpleural and posterobasal distribution [6]. At a later stage, fibroblast proliferation and activation lead to extracellular matrix deposition and fibrosis of the lung parenchyma, and there is a restrictive pattern with further declines in DLco and reductions in FVC [6]. HRCT exhibits a reticular pattern with intralobular opacity and thickening of the interlobular septa and, eventually, a honeycombing pattern with traction bronchiectasis [6].

Calprotectin is a heterodimer (S100A8/A9), playing a key role in innate immunity. It is primarily produced by neutrophils, but it is also found in other cells of the immune system, such as monocytes and macrophages or plasmacytoid dendritic cells, and can be released into the extracellular environment upon degranulation [7].

Calprotectin acts as an efficient damage-associated molecular pattern (DAMP), inducing inflammation by signaling through toll-like receptor 4 (TLR4) [7]. SSc epidermal explants release increased levels of S100A9, which was shown to induce fibroblast proliferation via TLR4, suggesting that the SSc epidermis provides an important source of proinflammatory S100A9 in SSc skin and, therefore, contributes to the inflammation seen in the disease [8]. It has been previously demonstrated that TLR4 signaling in SSc patients induces the production of transforming growth factor β (TGFβ), which triggers fibroblast proliferation, with consequent development of skin and pulmonary fibrosis [9]. Hesselstrand et al. demonstrated higher bronchoalveolar lavage fluid concentrations of S100A8/A9 in SSc patients than the HCs [10]. Recently, serum calprotectin has been proposed as a new biomarker for disease severity assessment in idiopathic pulmonary fibrosis (IPF) [11].

Due to its cytotoxic and proinflammatory characteristics, serum calprotectin regulates processes such as cell differentiation, proliferation, and the formation of neutrophil extracellular traps (NETs), and has been implicated in numerous rheumatic diseases [12,13]. Its levels are known to increase in the presence of systemic inflammation, making it an attractive marker for autoimmune diseases such as SSc.

Transcription levels of S100A8 and S100A9 in peripheral blood were found elevated in both SSc patients than healthy controls (HCs), and the plasma concentrations of S100A8 and S100A9 were significantly higher in dcSSc patients compared to the HCs [14]. Moreover, S100A8 and S100A9 levels were significantly increased in dcSSc patients with lung or kidney involvement [14].

It has been previously demonstrated that SSc patients have increased levels of fecal calprotectin, which strongly correlate with the presence of small intestinal bacterial overgrowth (SIBO) [15] and other markers of systemic inflammation, such as C-reactive protein (CRP) [16]. However, fecal calprotectin did not correlate with calprotectin levels in plasma [16].

The primary outcome of this study was to evaluate the serum calprotectin levels in SSc patients compared to HCs. The secondary outcomes of the study were to evaluate the possible correlation of serum calprotectin levels with clinical measures of disease activity and to investigate their possible association with microvascular and fibrotic complications of SSc, such as DUs and ILD.

## 2. Results

The median age of SSc patients enrolled was 58 years (IQR 50;65 years), and the median disease duration was 15 years (IQR 8;20). A total of 33 (44.6%) patients had dcSSc and 41 (55.4%) had limited cutaneous (lc)SSc, with a median mRSS of 12 (IQR 9;20). Most patients had Scl70 positivity (44.6%) and a late capillaroscopic pattern (48.6%). Median disease activity index (DAI) and disease severity scale (DSS) were 1.5 (IQR 0;3.26) and 5 (IQR 4;8), respectively. DUs were present in 13 (17.6%) patients, and ILD was diagnosed in 32 (43.2%) patients. The demographic and clinical features of SSc patients are shown in Table 1.

**Figure 1:**
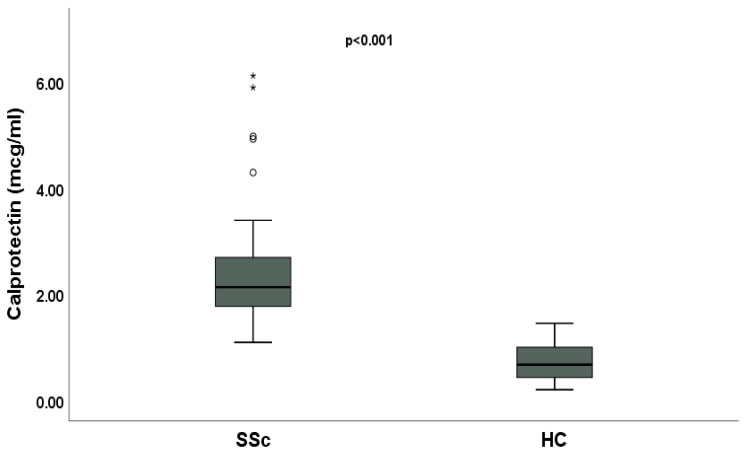
Comparative analysis of serum calprotectin between systemic sclerosis (SSc) patients and healthy controls (HCs). Asterisks and circles are outliers.

SSc patients had a statistically significantly higher median value of serum calprotectin than the HCs [2.13 mcg/mL (IQR 1.77;2.69) vs. 0.67 (IQR 0.43;1), *p* < 0.001] (Figure 1).

**Figure 2:**
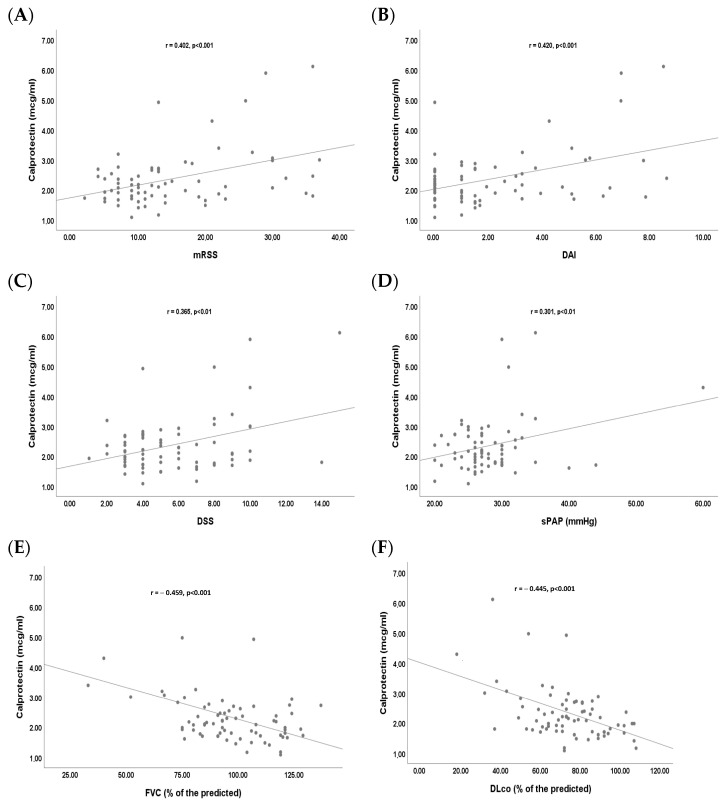
Bivariate linear correlation between serum calprotectin and disease variables in systemic sclerosis (SSc) patients. (**A**) Positive linear correlation between serum calprotectin and modified Rodnan skin score (mRSS); (**B**) positive linear correlation between serum calprotectin and disease activity index (DAI); (**C**) positive linear correlation between serum calprotectin and disease severity scale (DSS); (**D**) positive linear correlation between serum calprotectin and systolic pulmonary arterial pressure (sPAP); (**E**) negative linear correlation between serum calprotectin and forced vital capacity (FVC); (**F**) negative linear correlation between serum calprotectin and diffusion lung capacity for carbon monoxide (DLco).

We found a statistically significant positive linear correlation between serum calprotectin and mRSS (r = 0.402, *p* < 0.001), DAI (r = 0.420, *p* < 0.001), DSS (r = 0.365, *p* < 0.01) and sPAP (r = 0.301, *p* < 0.01) (Figure 2A–D). Moreover, we found a statistically significant negative linear correlation between serum calprotectin and FVC (r = −0.459, *p* < 0.001) and DLco (r = −0.445, *p* < 0.001) (Figure 2E,F).

We did not find any other statistically significant correlation between serum calprotectin and demographic or other clinical characteristics of the disease.

**Figure 3:**
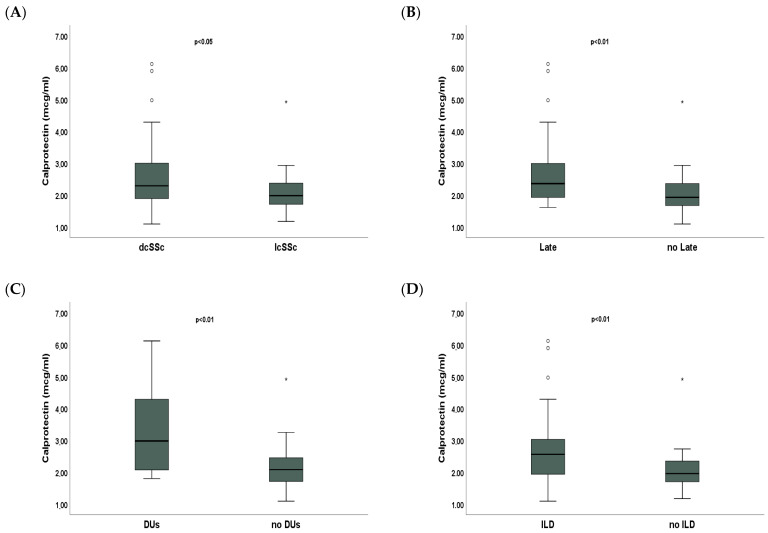
Comparative analysis of serum calprotectin according to disease variables in systemic sclerosis (SSc) patients. (**A**) Median serum calprotectin in diffuse cutaneous (dc)SSc and limited cutaneous (lc)SSc; (**B**) median serum calprotectin in SSc patients with a late capillaroscopic pattern and SSc patients with an early/active capillaroscopic pattern; (**C**) median serum calprotectin in SSc patients with and without digital ulcers (DUs); (**D**) median serum calprotectin in SSc patients with and without interstitial lung disease (ILD). Asterisks and circles are outliers.

dcSSc patients had statistically significantly higher median serum calprotectin than lcSSc patients [2.29 mcg/mL (IQR 1.89;3) vs. 1.98 mcg/mL (IQR 1.71;2.37), *p* < 0.05] (Figure 3A). SSc patients with a late capillaroscopic pattern had statistically significantly higher median serum calprotectin compared to patients with an early/active capillaroscopic pattern [2.36 mcg/mL (IQR 1.93;2.99) vs. 1.92 mcg/mL (IQR 1.67;2.36), *p* < 0.01] (Figure 3B). Median serum calprotectin was significantly higher in SSc patients with DUs than SSc patients without DUs [2.98 mcg/mL (IQR 2.07;4.29) vs. 2.08 mcg/mL (IQR 1.71;2.45), *p* < 0.01] (Figure 3C). SSc patients with ILD had statistically significantly higher median serum calprotectin compared to SSc patients without ILD [2.56 mcg/mL (IQR 1.94;3.03) vs. 1.96 mcg/mL (IQR 1.7;2.35), *p* < 0.01] (Figure 3D).

The multivariable stepwise logistic regression analysis showed serum calprotectin [OR 2.531 (CI 95%: 1.074;5.961), *p* < 0.05] and mRSS [OR 1.121 (CI 95%: 1.023;1.229), *p* < 0.05] as independently associated with DUs in SSc patients (Table 2). The multivariable stepwise logistic regression analysis showed serum calprotectin [OR 3.687 (CI 95%: 1.336;10.170), *p* < 0.05], FVC/DLco [OR 5.607 (CI 95%: 1.317;23.868), *p* < 0.05] and Scl70 [OR 13.744 (CI 95%: 3.650;51.752), *p* < 0.001] as independently associated with ILD in SSc patients (Table 2).

## 3. Discussion

In this monocentric cross-sectional study, SSc patients showed significantly higher levels of serum calprotectin than the HCs. Moreover, serum calprotectin correlates with disease severity and activity in SSc patients since it is independently associated with DUs and ILD.

Calprotectin is significantly higher in patients with SSc compared to the HCs, suggesting that increased levels of this protein reflect systemic inflammatory activity. This is in line with the knowledge that in SSc patients, inflammation plays a crucial role in activating the pathological mechanisms that lead to tissue damage, such as skin and lung fibrosis [17]. The initial inflammatory response, in fact, often precedes fibrotic damage. The correlation between inflammation and fibrosis is well documented in the literature, where it is known that inflammatory mediators, such as cytokines and growth factors (e.g., TGFβ), are active in the early stages of the disease and stimulate fibroblasts to proliferate and produce collagen, leading to fibrosis [17]. In this context, we may hypothesize that calprotectin released by activated immune cells promotes TLR4 signaling with consequent production of TGFβ, which triggers fibroblast proliferation, leading to skin and pulmonary fibrosis.

A moderate, statistically significant positive correlation was found between calprotectin and several measures of disease activity and severity, such as mRSS, DAI, and DSS, suggesting that calprotectin could be a marker of inflammation and progression of the disease. Moreover, patients with dcSSc have significantly higher levels of calprotectin than those with lcSSc. This is consistent with the hypothesis that increased serum calprotectin could be associated with more active and severe inflammation.

The results of the study show that patients with a late capillaroscopic pattern had higher calprotectin levels than those with an early/active pattern. This could suggest that calprotectin is not only linked to disease severity but that it could also be useful as a marker to monitor disease progression and transition from an active microvascular inflammatory phase to a fibrotic phase. The positive correlation between serum calprotectin and sPAP, although moderate, suggests that this protein could also be used as an early biomarker of the development or progression of pulmonary hypertension. Moreover, SSc patients with DUs had significantly higher levels of serum calprotectin compared to SSc patients without DUs. DUs are primarily the result of vascular disease and endothelial dysfunction, and local inflammation plays a critical role in their development and persistence, as activation of immune system cells in affected areas increases vascular damage and tissue necrosis [18]. Calprotectin appears to reflect the intensity of vascular inflammation and tissue damage and, therefore, may be useful not only for diagnosing DUs but also for predicting their evolution and monitoring the effectiveness of therapies.

Serum calprotectin also shows a moderate negative correlation with FVC and DLco, two key parameters for assessing lung function, suggesting a potential role for calprotectin as a marker of lung injury. Moreover, SSc patients with ILD show significantly higher levels of calprotectin, suggesting that systemic and pulmonary inflammation may be related. These findings suggest that calprotectin could be used as a biomarker to monitor the progression of pulmonary fibrosis in these patients.

Finally, in multivariable analysis, serum calprotectin was independently associated with DUs and ILDs, suggesting a possible role of this protein as a marker for these complications of SSc.

The present study has the following limitations: (i) it is a single-center study with a cross-sectional design; (ii) the sample size is relatively small since SSc is a rare disease; (iii) we did not measure fecal calprotectin, which could also provide insights into gastrointestinal involvement. It would be interesting to conduct longitudinal studies to test whether monitoring calprotectin levels over time can predict disease progression and the effectiveness of treatments. In addition, studies of the biological mechanisms linking calprotectin and tissue damage could lead to new therapeutic approaches. Despite this, we firmly believe that this study paves the way for new research toward a better understanding of the pathogenesis of SSc.

## 4. Materials and Methods

### 4.1. Subjects

In this monocentric cross-sectional study, we enrolled 74 consecutive SSc patients admitted to Policlinico Umberto I in Rome, who fulfilled the 2013 American College of Rheumatology/European League Against Rheumatism Collaborative Criteria (ACR/EULAR) for SSc [19]. Moreover, 50 HCs, matched for sex and age, were recruited among healthcare workers at the same tertiary hospital.

All SSc patients and HCs enrolled were under treatment with proton pump inhibitors.

Exclusion criteria were as follows: Concomitant or previous malignancy, infectious disease, or inflammatory bowel disease. Smokers, pregnant or breastfeeding women, and patients treated in the last 6 months with non-steroidal anti-inflammatory drugs (NSAIDs), immunosuppressive agents, or corticosteroids at an equivalent dose of prednisone ≥10 mg/day were also excluded.

The subjects’ written consent was obtained, and the study was conducted according to the Declaration of Helsinki. The study was approved by the Ethics Committee of Sapienza University of Rome (IRB n 0304).

### 4.2. Clinical Assessment

According to Le Roy et al. [20], the disease subset was defined as lcSSc or dcSSc, and the mRSS was assessed by the same experienced operator, blinded to laboratory assessment and other clinical characteristics of SSc patients. Disease duration (time from first non-Raynaud manifestation), DAI, and DSS were assessed following the European Scleroderma Trials and Research (EUSTAR) group indications [21,22]. NVC was performed on both hands at the level of the distal phalanx of the second, third, and fourth fingers using a videocapillaroscope equipped with a 200× magnification lens (VideoCap 3.0, DS Medica, Milano, Italy); the capillaroscopic patterns were classified as early, active, and late, according to Cutolo et al. [2]. NVC was performed after resting the subject in a temperature-controlled room at 24 ± 0.4 °C for 20 min by the same experienced operator, blinded to laboratory assessment and other clinical characteristics of SSc patients. DUs were defined according to Amanzi et al. [4]. Pulmonary function test (PFT) parameters, such as FVC and DLco, were recorded by the same experienced (and blinded) operator using a Quark PFT 2 spirometer (COSMED, Rome, Italy) and expressed according to the standards recommended by the American/European Respiratory Society [23]. HRCTs performed during the study or within the previous six months were evaluated by the same expert radiologist. ILD was defined as fibrotic changes affecting at least 10% of the lung parenchyma, according to a previous study [24].

### 4.3. Laboratory Assessment

Two serum aliquots from each patient were stored at −80 °C until use, and then tested for calprotectin serum using Aptiva’s particle-based multianalyte technology. Aptiva’s particle-based multi-analyte technology (PMAT) increases clinical confidence and diagnostic precision in the detection of autoantibodies and proteins [25]. Thawing of samples occurred once, with maintenance at room temperature, and immediate analysis thereafter. The analysis was conducted by an independent operator who was blinded to the clinical history of the samples, ensuring an unbiased assessment.

The assays were created by covalently binding antigens to paramagnetic microparticles, which carry a unique signal by an optical module. The optical module was composed of two light-emitting diode units set to different wavelengths and one charge-coupled device sensor. Particles were incubated with diluted patient samples, underwent a wash cycle, were incubated with human antiserum, and conjugated to a fluorescent probe. After another wash cycle, particles were analyzed using digital imaging technology installed in the platform [25].

### 4.4. Statistical Analysis

SPSS version 25.0 software (Bioz, Los Altos, CA, USA) was used for statistical analysis. After the evaluation of normality, continuous variables were expressed as median and interquartile range (IQR), while categorical variables were expressed as absolute frequency and percentage (%). Student’s *t*-test or Mann-Whitney’s U-test was used to evaluate differences between groups, as appropriate. Bonferroni’s corrections were applied in cases of multiple comparisons. The chi-square or Fisher’s exact test was used to evaluate differences between categorical variables, as appropriate. The 2-tailed Pearson or Spearman’s correlation test was used for bivariate correlations. Stepwise logistic regression analysis was used to evaluate the association between a dependent dichotomous variable (ILD or DU) and continuous (calprotectin) or dichotomous (FVC/DLco and Scl70 for ILD; mRSS and late pattern for DUs) independent variables, which were significant at the bivariate analysis. Results were expressed as odds ratios (ORs) with 95% confidence intervals (CIs). A *p*-value <0.05 was considered significant.

## 5. Conclusions

In conclusion, our data show that serum calprotectin is higher in SSc patients with DUs or ILD. These associations are potential candidates for further investigation into the ability of serum calprotectin to predict the development of DUs or ILD in SSc patients. Further studies may provide valuable insights into the underlying mechanisms of the complications related to SSc and help to identify new treatment strategies.

## Figures and Tables

**Table 1:** Demographic and clinical features of systemic sclerosis (SSc) patients.

Age, years, median, and IQR	58 (50;65)
Female, n (%)	63 (85.1)
dcSSc, n (%)	33 (44.6)
Disease duration, years, median and IQR	15 (8;20)
mRSS, median and IQR	12 (9;20)
SSc-specific autoantibodies	
Anti-topoisomerase I, n (%)	33 (44.6)
Anti-centromere, n (%)	19 (25.7)
Anti-RNA polymerase III, n (%)	1 (1.3)
None, n (%)	21 (28.4)
NVC	
Early, n (%)	17 (23)
Active, n (%)	21 (28.4)
Late, n (%)	36 (48.6)
DAI, median and IQR	1.5 (0;3.26)
DSS, median and IQR	5 (4;8)
sPAP, mmHg, median and IQR	27 (25;30)
FVC, % of the predicted, median and IQR	94.5 (83;112)
DLco, % of the predicted, median and IQR	73 (63;86)
ILD, n (%)	32 (43.2)
New DUs, n (%)	13 (17.6)
PAH, n (%)	3 (4)

SSc: systemic sclerosis; dcSSc: diffuse cutaneous systemic sclerosis; mRSS: modified Rodnan skin score; NVC: nailfold videocapillaroscopy; DAI: disease activity index; DSS: disease severity scale; sPAP: systolic pulmonary arterial pressure; FVC: forced vital capacity; DLco: diffusion lung capacity for carbon monoxide; DUs: digital ulcers; PAH: pulmonary arterial hypertension; IQR: interquartile range.

**Table 2:** Multivariable stepwise logistic regression analysis showing the association between interstitial lung disease (ILD) or digital ulcers (DUs) and independent variables.

**ILD**		
	**OR (CI 95%)**	***p***
Calprotectin, mcg/mL	3.687 (1.336;10.170)	<0.05
FVC/DLco	5.607 (1.317;23.868)	<0.05
Scl70	13.744 (3.650;51.752)	<0.001
**DUs**		
	**OR (CI 95%)**	***p***
Calprotectin, mcg/mL	2.531 (1.074;5.961)	<0.05
Late	1.701 (0.236;12.250)	>0.05
mRSS	1.121 (1.023;1.229)	<0.05

ILD: interstitial lung disease; DUs: digital ulcers; FVC: forced vital capacity; DLco: diffusion lung capacity for carbon monoxide; mRSS: modified Rodnan skin score; OR: odds ratio; CI: confidence interval.

## Data Availability

All data are present in the manuscript.

## Abbreviations

The following abbreviations are used in this manuscript:

ACR/EULAR	American College of Rheumatology/European League Against Rheumatism Collaborative Criteria
CI	confidence interval
CRP	C-reactive protein
DAMP	damage-associated molecular pattern
DAI	disease activity index
(dc)SSc	diffuse cutaneous systemic sclerosis
DLco	diffusion lung capacity for carbon monoxide
DSS	disease severity scale
DUs	digital ulcers
EUSTAR	European Scleroderma Trials and Research
FVC	forced vital capacity
HCs	healthy controls
HRCT	high-resolution computed tomography
ILD	interstitial lung disease
IPF	idiopathic pulmonary fibrosis
IQR	interquartile range
(lc)SSc	limited cutaneous systemic sclerosis
mRSS	modified Rodnan skin score
NETs	neutrophil extracellular traps
NSAIDs	non-steroidal anti-inflammatory drugs
NVC	nailfold videocapillaroscopy
OR	odds ratio
PFTs	pulmonary function tests
PMAT	particle-based multi-analyte technology
SIBO	small intestinal bacterial overgrowth
sPAP	systolic pulmonary artery pressure
SSc	systemic sclerosis
TGF β	transforming growth factor β
TLR4	toll-like receptor 4
